# Une tachycardie à QRS large mal tolérée chez un nourrisson

**DOI:** 10.11604/pamj.2017.27.157.10364

**Published:** 2017-06-30

**Authors:** Désiré Alain Affangla, Mohamed Leye, Angèle Wabo Simo, Franck D’Almeida, Thérèse Yandé Sarr, Adamson Phiri, Adama Kane

**Affiliations:** 1 Hôpital Saint Jean de Dieu, Thiès, Sénégal; 2 UFR Sciences de la Santé, Université de Thiès, Sénégal; 3 Centre Hospitalier Régional de Thiès, Sénégal; 4 Hôpital Bartimée, Thiès, Sénégal; 5 UFR Sciences de la Santé, Université Gaston Berger de Saint Louis, Sénégal

**Keywords:** Trouble du rythme, tachycardie, nourrisson, cardioversion électrique, Sénégal, Rhythm disorder, tachycardia, newborn, electrical cardioversion, Senegal

## Abstract

Les tachycardies à QRS large mal tolérées du nourrisson posent le problème de leur diagnostic et de la prise en charge en urgence. Nous rapportons un cas de tachycardie à QRS large chez un nourrisson de 35 jours reçu pour détresse cardio-circulatoire. Le cœur était morphologiquement normal à l’échographie cardiaque Doppler. Un traitement par une dose charge d’Amiodarone n’a pas permis de réduire cette tachycardie. Un retour en rythme sinusal a été obtenu après cardioversion par un défibrillateur externe semi-automatique type Lifeline. Un traitement d’entretien par Amiodarone per os est institué et le patient est en rythme sinusal à 03 mois.

## Abstract (en)

Poorly tolerated broad QRS complex tachycardia in a newborn poses problems with its diagnosis and emergency management. We report the case of a 35-day-old newborn with broad QRS complex tachycardia admitted because of cardiocirculatory distress. Doppler echocardiography showed morphologically normal heart. The patient received a loading dose of amiodarone but it didn’t attenuate tachycardia. Normal sinus rhythm was restored after cardioversion through Lifeline semi-automatic external defibrillator. Maintenance therapy was based on oral amiodarone. The patient had normal sinus rhythm at 03 months of follow-up.

## Introduction

Les troubles du rythme cardiaques mal tolérés du nourrisson posent le problème de leur diagnostic et de leur prise en charge en urgence particulièrement dans les hôpitaux n’ayant pas de service spécialisé de cardiopédiatrie. Nous rapportons un cas d’une tachycardie à QRS large mal tolérée chez un nourrisson.

## Patient et observation

**Figure 1:**
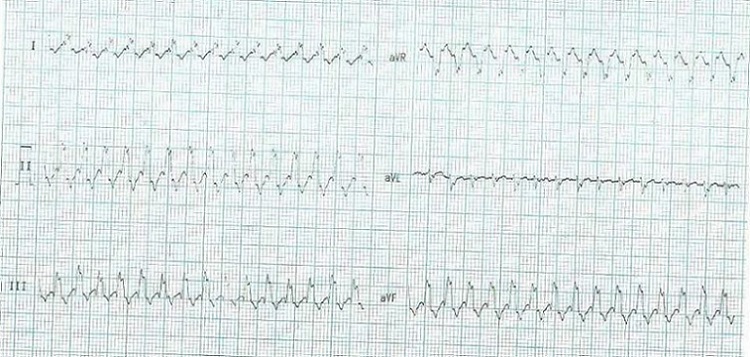
Tachycardie à QRS large

Il s’agit d’un nourrisson de sexe féminin âgé de 35 jours reçu en urgence pédiatrique pour une difficulté respiratoire évoluant depuis 24 heures accompagnée d’un geignement et d’une toux. On ne notait pas de fièvre ni de diarrhée. La mère âgée est âgée de 18 ans primipare et primigeste. Le déroulement de la grossesse était sans particularité. L’accouchement à terme 38 semaine d’aménorrhée était eutocique par voie basse avec un Apgar = 10 à la première minute. Le poids de naissance était de 3300 g. On ne notait pas de tare familiale. L’examen du nourrisson relevait une polypnée superficielle avec un score de Silverman = 4 : un balancement thoraco-abdominal, un battement des ailes du nez et un geignement. Il n’y avait pas de cyanose et la SpO2 à l’air libre était à 96%. Les champs pulmonaires étaient libres et l’auscultation cardiaque relevait une tachycardie régulière très rapide à 250 minutes. L’abdomen était souple avec la présence d’une hépatomégalie homogène avec une flèche hépatique = 14 cm sur la ligne médio claviculaire. Les mensurations pour l’âge étaient normale avec un périmètre crânien = 38cm; un périmètre brachial = 13 cm; un poids= 4700 g; une taille= 62 cm. Un électrocardiogramme (ECG) standard à 12 dérivations relevait une tachycardie régulière à QRS large avec une fréquence ventriculaire de 300/minutes (Figure 1). L’échographie Doppler cardiaque montrait un cœur très rapide avec une minime insuffisance mitrale et une légère dilatation de l’oreillette gauche sans autres anomalies. La radiographie thoracique montrait une cardiomégalie modérée avec un rapport cardio- thoracique = 0.65. Le bilan biologique effectué retrouvait : une hyperleucocytose à 18130/mm3 à prédominance de polynucléaire neutrophile, une anémie avec un taux d’hémoglobine à 10,2g/dl normochrome normocytaire ; la CRP normale <6 mg, un ionogramme sanguin normal. Au total: il s’agissait d’une défaillance cardiaque aigue sur tachycardie.

**Figure 2:**
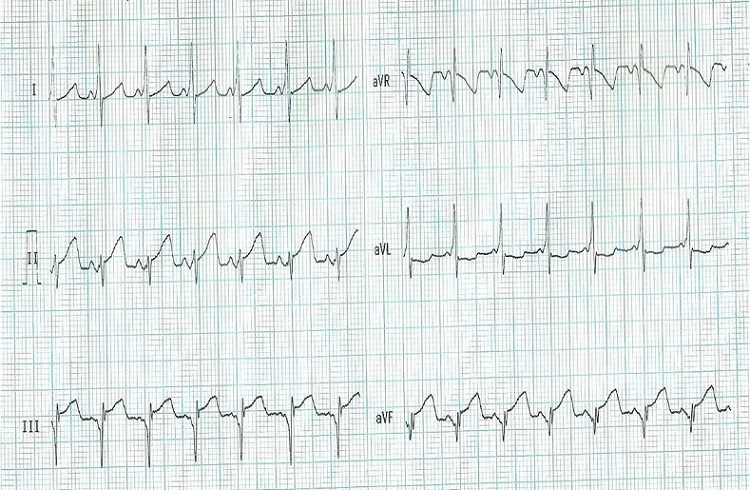
Rythme sinusal avec PR court et onde delta

Au plan thérapeutique une dose charge d’Amiodarone à la dose de 10 mg/Kg n’a pas permis de réduire cette tachycardie. La patiente à reçu également du Furosémide à la dose 2mg/Kg/jour et du Ceftriaxone à la dose 75 mg/Kg/jour. Le lendemain, devant la persistance des signes d’insuffisance cardiaque, une cardioversion électrique par un défibrillateur externe semi-automatique type Lifeline adulte est effectuée avec un retour à un rythme sinusal stable suivie d’une nette amélioration marquée par la régression immédiate de la dyspnée et de l’hépatomégalie en quelques jours. Sur le tracé ECG en rythme sinusal (Figure 2), on pouvait voir un aspect de PR court avec onde delta permettant d’évoquer les diagnostics suivants : une tachycardie antidromique sur Wolf Parkinson White (WPW) postéro septal gauche, une tachycardie ventriculaire et une tachycardie supra ventriculaire avec aberration de conduction. La mise en exéat est faite avec un maintient de l’Amiodarone en traitement d’entretien à la dose de 5mg/Kg/j.

## Discussion

Les tachycardies mal tolérées diagnostiquées chez le nourrisson sont rares. Le diagnostic de tachycardie à complexes larges est retenu chez l’enfant quand la durée des QRS excède 80 ms et toute tachycardie à complexes larges doit être considérée comme une tachycardie ventriculaire (TV) jusqu’à preuve du contraire et prise en charge rapidement [1]. Chez notre patiente le trouble du rythme était grave, responsable d’une insuffisance cardiaque globale aigue. La détérioration de fonction systolique ventriculaire gauche est une modalité évolutive [2]. La fonction systolique chez notre patiente était encore normale témoignant sans doute de la précocité du diagnostic. La non réduction de cette tachycardie aurait certainement abouti soit à une mort subite ou une déchéance de la fonction systolique ventriculaire gauche réalisant une myocardiopathie rythmique [3].

Le traitement en urgence requiert l’utilisation de différents antiarythmiques ou d’une cardioversion électrique [4–9]. Nous avons opté pour la cardioversion pharmacologique du fait de la non disponibilité de défibrillateur pédiatrique. En première intention, les ?-bloquants oraux (notamment le Sotalol) seuls ou associés aux agents anti arythmiques de la classe IC sont préconisés en l’absence de contre indication [4]. Devant la non disponibilité de ces agents antiarythmiques nous avons administré l’amiodarone qui est indiquée dans la prise en charge des tachycardies supraventriculaires symptomatiques [5, 10, 11]. Nous avons administré une dose charge d’Amiodarone 500 mg/m² qui n’a permis de réduire la tachycardie. Une cardioversion électrique a été effectuée avec défibrillateur sémi-automatique faute d’un défibrillateur externe avec palettes pédiatriques.

Le traitement de choix d’un patient en détresse circulatoire du fait d’une Tachycardie à QRS large est la cardioversion par choc électrique externe. Le choc doit être “synchronisé” car un choc sur l’onde T peut faire dégénérer la TV en fibrillation ventriculaire (FV). Son énergie est de 2 à 3 joules par kg de poids. Le choc électrique externe a l’avantage d’être efficace sans être pro-arythmogène, ses inconvénients sont de nécessiter une sédation et, si les chocs sont répétés, d’altérer la fonction myocardique. L’analyse de l’ECG en rythme sinusal chez notre patiente montre un PR court à 8/100 sec et une onde delta faisant évoquer le diagnostic d’une tachycardie antidromique sur Wolf Parkinson White (WPW) postéro septal gauche, une tachycardie ventriculaire et une tachycardie supra ventriculaire avec aberration de conduction. Nous avons éliminé le diagnostic de tachycardie antidromique sur Wolf Parkinson White parce que les ondes delta ne se voient pas dans les dérivations DIII, avF et précordiales antérieures sur les QRS en tachycardie. Les deux autres hypothèses restant plausibles étant la tachycardie ventriculaire monomorphe et la tachycardie supra ventriculaire avec aberration de conduction.

Le traitement pour le maintien en rythme sinusal par les agents antiarytmique est délicat chez le nourrisson. L’amiodarone est assez bien tolérée mais des effets secondaires notamment une dysthyroïdie, une fibrose pulmonaire ou une épididymite peuvent être observées [12, 13]. Notre patiente ne présente aucun effet indésirable à 3 mois de suivi.

## Conclusion

La tachycardie peut être très mal tolérée chez le nourrisson. Le pronostic immédiat dépend de la capacité à réduire la tachycardie par cardioversion électrique alors que les structures sanitaires au Sénégal disposant d’un défibrillateur pédiatriques sont rares. Le traitement arythmique par amiodarone permet le maintien en rythme sinusal.

## Conflits d’intérêts

Les auteurs ne déclarent aucun conflit d'interêts.
